# Combining gene expression microarrays and Mendelian randomization: exploring key immune-related genes in multiple sclerosis

**DOI:** 10.3389/fneur.2024.1437778

**Published:** 2024-11-27

**Authors:** Shuangfeng Ding, Yunyun Zhang, Yunzhe Tang, Ying Zhang, Mingyuan Liu

**Affiliations:** ^1^Yueyang Hospital of Integrated Traditional Chinese and Western Medicine, Shanghai University of Traditional Chinese Medicine, Shanghai, China; ^2^Department of Neurology, Qingpu Branch of Zhongshan Hospital, Fudan University, Shanghai, China

**Keywords:** multiple sclerosis, bioinformatics analysis, protein–protein interaction, Mendelian randomization, gene expression microarrays

## Abstract

**Objective:**

Multiple Sclerosis (MS) is an autoimmune disorder characterized by demyelination occurring within the white matter of the central nervous system. While its pathogenesis is intricately linked with the body’s immune response, the precise underlying mechanisms remain elusive. This study aims to explore potential immune-related genes associated with MS and assess the causal relationship between these genes and the risk of developing MS.

**Methods:**

We retrieved expression datasets of peripheral blood mononuclear cells from MS patients from the Gene Expression Omnibus (GEO) database. Immune-related differentially expressed genes (IM-DEGs) were identified using the ImmPort database. GO and KEGG analyses were subsequently performed to elucidate the functions and pathways associated with the IM-DEGs. To visualize protein–protein interactions (PPIs), we used STRING, Cytoscape, and Cytohubba to construct networks of PPIs and hub genes. The diagnostic efficacy of hub genes was assessed using the nomogram model and ROC curve. The correlation of these hub genes was further validated in the mouse EAE model using quantitative PCR (qPCR). Finally, Mendelian randomization (MR) was performed to ascertain the causal impact of hub genes on MS.

**Results:**

Twenty-eight IM-DEGs were selected from the intersection of DEGs and immune genes. These genes are involved mainly in antigen receptor-mediated signaling pathways, B cell differentiation, B cell proliferation, and B cell receptor signaling pathways. Using Cytoscape software for analysis, the top 10 genes with the highest scores were identified as PTPRC, CD19, CXCL8, CD79A, IL7, CR2, CD22, BLNK, LCN2, and LTF. Five hub genes (PTPRC, CD19, CXCL8, CD79A, and IL7) are considered to have strong diagnostic potential. In the qPCR validation, the relative expression of these five genes showed significant differences between the control and EAE groups, indicating that these genes may play a potential role in the pathogenesis of MS. The MR results indicate that elevated levels of CD79A (OR = 1.106, 95% CI 1.002–1.222, *p* = 0.046) are causally positively associated with the risk of developing MS.

**Conclusion:**

This study integrated GEO data mining with MR to pinpoint pivotal immune genes linked to the onset of MS, thereby offering novel strategies for the treatment of MS.

## 1 Introduction

Multiple Sclerosis (MS) is an immune-mediated inflammatory demyelinating disease of the central nervous system, characterized by lesions that exhibit both temporal and spatial dissemination features (1, 2). This condition primarily affects young adults aged 20–40, with a higher prevalence among females. The incidence and prevalence of this condition are increasing in both developed and developing countries (3). The etiology of MS remains unclear, but it is widely believed to involve a combination of factors including viral infection, genetic susceptibility, environmental factors, lifestyle, and immune dysregulation. The core mechanism involves the loss of self-immune tolerance by T cells, which primarily target sites such as the optic nerve, spinal cord, brainstem, and periventricular white matter. This leads to demyelination and loss of oligodendrocytes in the brain and spinal cord, ultimately resulting in disease onset (4, 5). Currently, both domestic and international treatments for MS primarily involve the use of corticosteroids and immunomodulators. Although these interventions can effectively alleviate symptoms, they are associated with high costs, significant side effects, and limited efficacy, leaving patient prognosis still concerning (6). Therefore, it is essential to identify new critical targets and analyze the molecular mechanisms underlying the disease pathogenesis, in order to provide new avenues for the treatment of MS.

In recent years, there have been significant advances in molecular-level research across various fields of disease, such as cancer, immune system disorders, and cardiovascular diseases. These advances have been made possible by the establishment of high-throughput sequencing platforms for gene chips. This technology has not only expanded our understanding of the underlying pathogenic mechanisms but has also become an important method and tool for screening important genetic materials during disease occurrence, identifying diseases, and predicting prognosis (7).

Mendelian randomization (MR) is a widely employed methodology that leverages Mendelian inheritance of genetic variations as instrumental variables for evaluating causal relationships between exposure factors and diseases. This approach is extensively utilized for probing the causal links between clinical factors and diseases (8, 9). Due to the random nature of genetic variations, MR analysis can provide reliable analytical results by reducing potential confounding factors and analyzing the correlation between exposure factors and outcome factors from a genetic perspective (10).

Several studies have employed MR in conjunction with genomic or proteomic techniques to identify multiple potential therapeutic targets (11–13). These findings underscore the effectiveness of gene chip technology, genome-wide association studies, and bioinformatics analysis methods as viable approaches for elucidating the pathogenesis of diseases such as MS.

This study conducted bioinformatic analysis of gene expression microarray data obtained from public databases to construct gene networks and identify potential key molecular targets related to immunity. Additionally, qPCR validation confirmed the relevance of these genes in the pathogenesis of MS. Finally, through MR analysis, the causal relationship between these hub genes and MS was inferred, which may provide new insights into the pathogenesis and clinical treatment of MS.

## 2 Materials and methods

### 2.1 Data collection

Using the search terms “Multiple Sclerosis” and “*Homo sapiens*,” we accessed the Gene Expression Omnibus (GEO) database^1^ to obtain the publicly available gene expression profile GSE21942 (14). The dataset includes gene expression array data of peripheral blood mononuclear cells from 14 MS patients and 15 healthy individuals. The mean age of the patients was 54.2 years, while the control group had a mean age of 71.6 years.

### 2.2 Identification of differentially expressed genes

We preprocessed the selected dataset using R software (version 4.3.2), including batch correction and normalization. Subsequently, we employed the “limma” package (15) to screen for DEGs in the GSE21942 dataset, setting *p* < 0.05 and a |log2-fold change (FC)| ≥ 1 as the identification thresholds. After analyzing the significantly differentially expressed genes, we employed the “pheatmap” and “ggplot2” R packages to create volcano plots and heatmaps illustrating the DEGs. Afterward, we downloaded an immune gene list from the ImmPort database^2^ and intersected it with the set of DEGs to obtain IM-DEGs for further analysis.

### 2.3 Functional enrichment and pathway analysis of candidate hub genes

We performed annotation and visualization analysis of the IM-DEGs using the enrichGO and enrichKEGG functions from the “ClusterProfiler” R package. GO is utilized to classify the functions of gene products, including biological processes (BP), molecular functions (MF), and cellular components (CC) (16). KEGG (17) was used to explore relevant pathways and advanced biological functions. We selected the most significantly enriched functions among the IM-DEGs for sorting and analysis.

### 2.4 Identification of hub genes

We utilized the Search Tool for the Retrieval of Interacting Genes/Proteins (STRING) database^3^ to construct a protein–protein interaction (PPI) network of the IM-DEGs, with a confidence score threshold set at >0.4, and hidden genes that were not connected. Subsequently, we visualized the network using Cytoscape 3.10.1 software. Then, employing the CytoHubba plugin within Cytoscape, we identified the top 10 hub genes using the MCC algorithm (18).

### 2.5 Nomogram model and ROC curve

Next, we constructed a nomogram model and ROC curves to evaluate the diagnostic efficacy of the top five genes (PTPRC, CD19, CXCL8, CD79A, and IL7). The nomogram is based on multivariable regression analysis and is primarily used to integrate multiple predictive factors onto a single plane, visually expressing the relationships among the variables in the predictive model. The nomogram model plays a significant role in supporting clinical decision-making, such as aiding in the early identification of high-risk patients and developing personalized treatment plans. The ROC curve serves as a binary classification model, where the output of diagnostic tests is categorized into a clearly defined binary outcome (e.g., presence or absence of disease), making it widely applicable for assessing the performance of different classification methods. In this study, we extracted the expression matrix and grouping information (disease status: 1 for patients and 0 for healthy controls) for these five hub genes from the GSE21294 dataset, and conducted data analysis and visualization using the “rms” and “ROC” software packages (19). The nomogram calculates a total score based on the upregulation or downregulation of these five genes in the samples, thereby assessing the risk of MS. The ROC curve evaluates the classification and diagnostic performance of these hub genes for distinguishing between patients and healthy individuals by calculating the area under the curve (AUC); an AUC value closer to 1 reflects higher accuracy of the predictive model.

### 2.6 Establishment of the EAE mouse model

Twelve 8-week-old female C57BL/6 mice, weighing 18–20 g and maintained under SPF conditions, were acclimatized to the barrier environment for 2 weeks prior to the experiment. On the day of induction, the mice were anesthetized with 1% sodium pentobarbital (30 mg/kg) and randomly assigned to either the control group or the EAE group, with an equal number of mice in each group. A total of 4 mg of myelin oligodendrocyte glycoprotein 35–55 (MOG_35-55_, Pufei Biotechnology) was dissolved in PBS, and 10 mg of H37Ra (BD) was dissolved in incomplete Freund’s adjuvant (IFA, Sigma). The two solutions were emulsified into a white, opaque suspension. The emulsified mixture was subcutaneously injected at two sites on the dorsal side of each mouse at a volume of 10 mL/kg. Additionally, on the day of induction and the following day, each mouse was intraperitoneally injected with pertussis toxin (PTX, Difco) at a volume of 10 mL/kg.

### 2.7 Real-time quantitative polymerase chain reaction

Four weeks post-induction, mice were euthanized, and their brain tissues were harvested. Total RNA was extracted from tissue homogenates following the manufacturer’s protocol (EZBioscience). The RNA concentration was measured, and cDNA was synthesized using the Color Reverse Transcription Kit with gDNA Remover (EZBioscience). Quantitative PCR (qPCR) was performed using the 2× Color SYBR Green qPCR Master Mix (ROX2) (EZBioscience) on a 7,500 Real-Time PCR System (Applied Biosystems). Relative gene expression levels were calculated using the 2^−ΔΔCt^ method. Primers were synthesized by Sangon Biotech, and their sequences are provided in Supplementary Table S1.

### 2.8 Mendelian randomization

To explore the causal relationship between the selected hub genes and MS, we used the “TwoSample” software package to conduct a two-sample MR analysis. The MR analysis utilizes genetic variations as instrumental variables (IVs) to explore the causal relationship between exposure and outcome, which can reduce the interference of confounding factors. There are three assumptions for the screening of IVs: first, the IV has a strong correlation with the exposure; second, the IV can only affect the outcome through the exposure; third, the IV is independent of confounding factors. In this study, the data used for MR analysis were obtained from the publicly available Genome-Wide Association Study (GWAS) database (https://gwas.mrcieu.ac.uk/). The selected single nucleotide polymorphisms (SNPs) were defined as instrumental variables (IVs), with the specific dataset detailed in Table 1. After obtaining the available IVs according to the screening conditions, the inverse variance weighted (IVW), MR-Egger, weighted median, simple mode, and weighted mode methods were used to evaluate the causal association between the hub genes and MS. The results of IVW will be regarded as the main conclusion because this method is the most effective MR analysis method. A sensitivity analysis was also conducted, whereby the removal of any single SNP resulted in consistent positive or negative outcomes, indicating that the results were not influenced by any particular outlier.

**Table 1:** The GWAS data information for the exposure factors and outcome variables.

Dataset	Category	Number of SNPs	Sample size
ieu-b-18 (outcome)	MS	6,304,359	115,803
ebi-a-GCST90002041 (exposure)	PTPRC	14,155,839	1,635
prot-a-1543 (exposure)	IL7	10,534,735	3,301
prot-a-461 (exposure)	CD79A	10,534,735	3,301
ebi-a-GCST90002028 (exposure)	CD19	14,903,706	3,112
ebi-a-GCST90011994 (exposure)	CXCL8	12,717,989	21,758

### 2.9 Statistical methods

Data analysis and graphical representation were performed using GraphPad Prism 9.4.1 software. A *p*-value of less than 0.05 was considered statistically significant.

## 3 Results

### 3.1 Identification of differentially expressed genes

**Figure 1:**
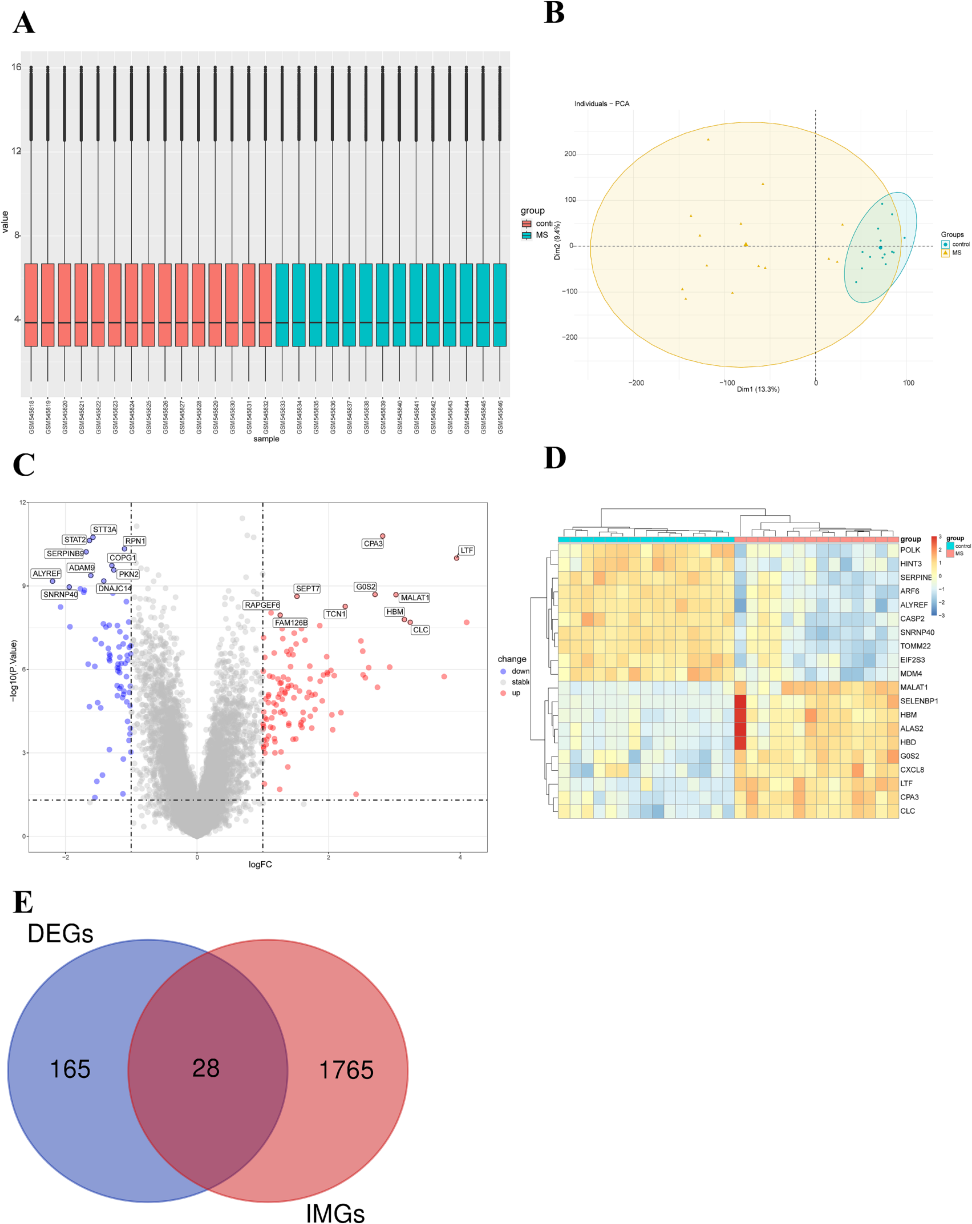
Expression microarray dataset of MS patients. **(A)** Boxplot of the expression microarray dataset of MS patients. **(B)** Principal component analysis of the expression microarray dataset of MS patients. **(C)** Volcano plot of differentially expressed genes. **(D)** Heatmap of the top 10 upregulated and downregulated genes with the smallest *p-*values. **(E)** Venn diagram of DEGs and immune gene sets.

After obtaining the MS dataset (GSE21942), we standardized the data and found good uniformity, indicating comparability between the two groups (Figure 1A). The results of the principal component analysis revealed considerable differences between the centers of the MS group and the control group, indicating gene expression differences between the two groups (Figure 1B). Using |logFC| ≥ 1 and *p* < 0.05 as the thresholds, a total of 193 DEGs were selected (110 upregulated and 76 downregulated) (Supplementary Table S2). The volcano plot and heatmap displayed the top 10 significantly differentially expressed upregulated and downregulated genes (Figures 1C, D). The top 10 upregulated genes with the smallest *p* values in the MS group and the control group were CPA3, LTF, GOS2, MALAT1, SEPT7, TCN1, RAPGEF6, FAM126B, HBM, and CLC. The top 10 downregulated genes with the highest frequency were STT3A, STAT2, RPN1, SERPINB9, COPG1, PKN2, ADAM9, DNAJC14, ALYREF, and SNRNP40. Simultaneously, 1,793 immune-related genes were retrieved from the ImmPort database. By intersecting the 193 DEGs with the 1,793 immune-related genes (Supplementary Table S3), we ultimately obtained 28 IM-DEGs (Figure 1E).

### 3.2 GO and KEGG analyses

**Figure 2:**
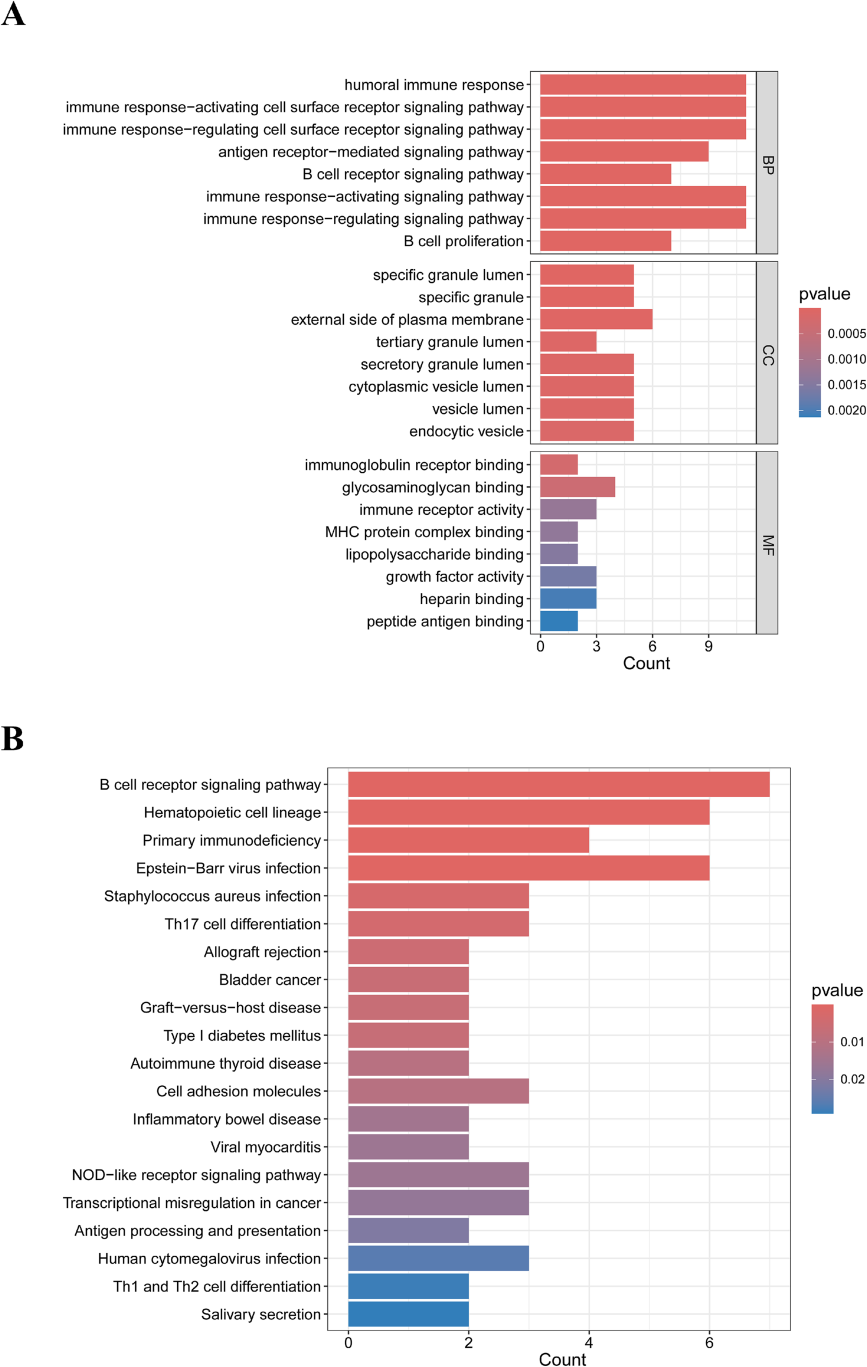
Results of the biological processes and signaling pathway analysis of IM-DEGs. **(A)** GO enrichment analysis of IM-DEGs. **(B)** KEGG enrichment analysis of IM-DEGs.

The GO enrichment analysis of IM-DEGs was primarily conducted using the clusterProfiler package in R, providing standardized descriptions of the differential genes, including their involvement in biological pathways, functions, and cellular localization. Figure 2A displays the top 8 results of the GO enrichment analysis for the 28 DEGs. The study revealed that these targets are primarily involved in biological processes such as the humoral immune response, immune response-activating cell surface receptor signaling pathway, and immune response-regulating cell surface receptor signaling pathway. The genes were mainly localized in the specific granule lumen and on the external side of the plasma membrane, while their molecular functions included immunoglobulin receptor binding, glycosaminoglycan binding, and immune receptor activity. Additionally, KEGG enrichment analysis indicated that these genes mainly influence biological pathways such as the B cell receptor signaling pathway, hematopoietic cell lineage, primary immunodeficiency, and Epstein–Barr virus infection (Figure 2B). In summary, the functions of IM-DEGs are closely associated with immunity.

### 3.3 Establishment of the PPI network and identification of hub genes

**Figure 3:**
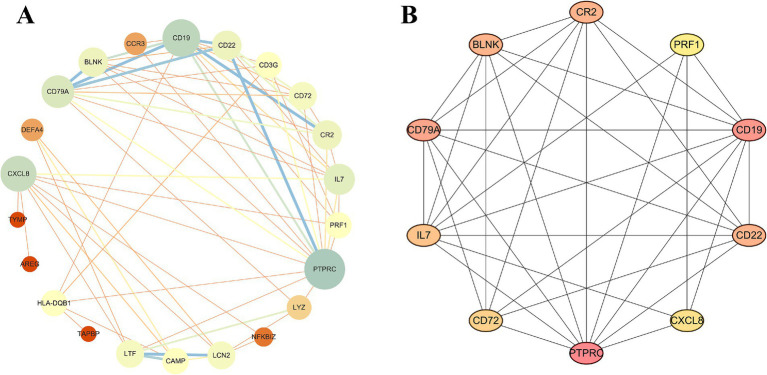
Construction of the PPI network. **(A)** PPI network of IM-DEGs. **(B)** Core genes were obtained from the interaction network using the MCC algorithm.

We utilized the STRING online tool to construct a PPI network consisting of 28 genes. After unconnected nodes were hidden, the resulting network comprised 22 nodes and 64 edges. Subsequently, visualization was performed using Cytoscape software (Figure 3A). Next, we employed the MCC algorithm to select the top 10 ranked genes, namely, PTPRC, CD19, CXCL8, CD79A, IL7, CR2, CD22, BLNK, LCN2, and LTF (Figure 3B), where darker colors indicate higher node scores.

### 3.4 Risk prediction model and experimental validation

**Figure 4:**
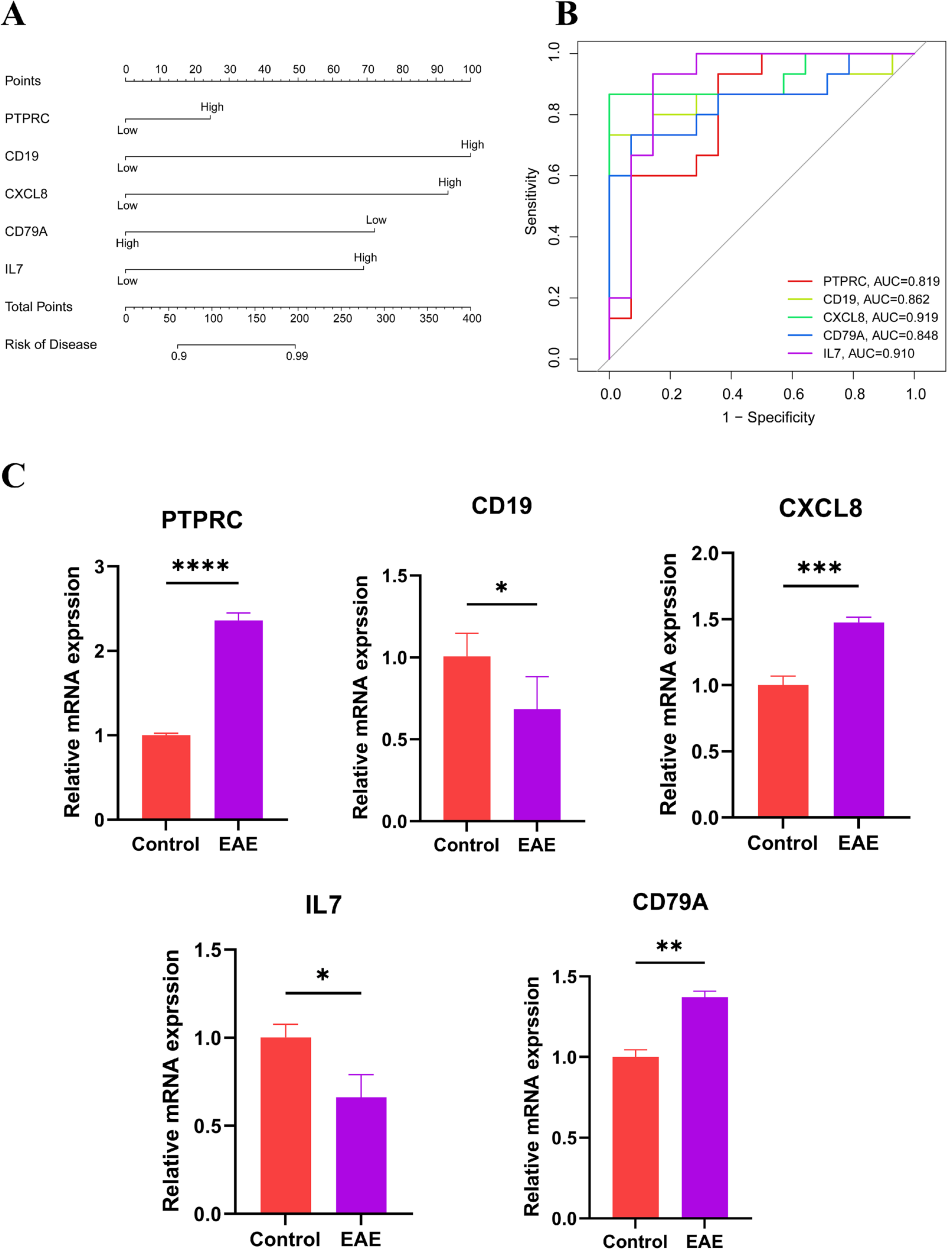
Risk assessment of MS. **(A)** Nomogram: scores are assigned based on the expression levels of individual genes, and the total score is obtained by summing the scores of each independent variable. The total score is then used to estimate the risk of developing MS. **(B)** ROC curve: to evaluate the ability of hub genes to distinguish between MS patients and healthy controls; an AUC value closer to 1 indicates higher diagnostic value. **(C)** Expression levels of the hub genes in normal mice and EAE models (*p < 0.05, **p < 0.01, ***p < 0.001, ****p < 0.0001).

We established a nomogram model comprising five central genes (PTPRC, CD19, CXCL8, CD79A and IL7) to predict the risk of MS (Figure 4A). The results demonstrate that these genes perform well in predicting MS risk. Subsequently, we calculated the ROC curves for these genes to evaluate their diagnostic performance. The results indicate that the AUC values of PTPRC, CD19, CXCL8, CD79A, and IL7 effectively distinguish MS patients from the control group (Figure 4B), with values of 0.819, 0.862, 0.919, 0.848, and 0.910, respectively. Then, we verified the expression levels of these five hub genes in the control group and the EAE group through qPCR experiments. The results indicated that there were significant differences in these genes between the two groups (Figure 4C).

### 3.5 CD79A was causally associated with the risk of MS

**Figure 5:**
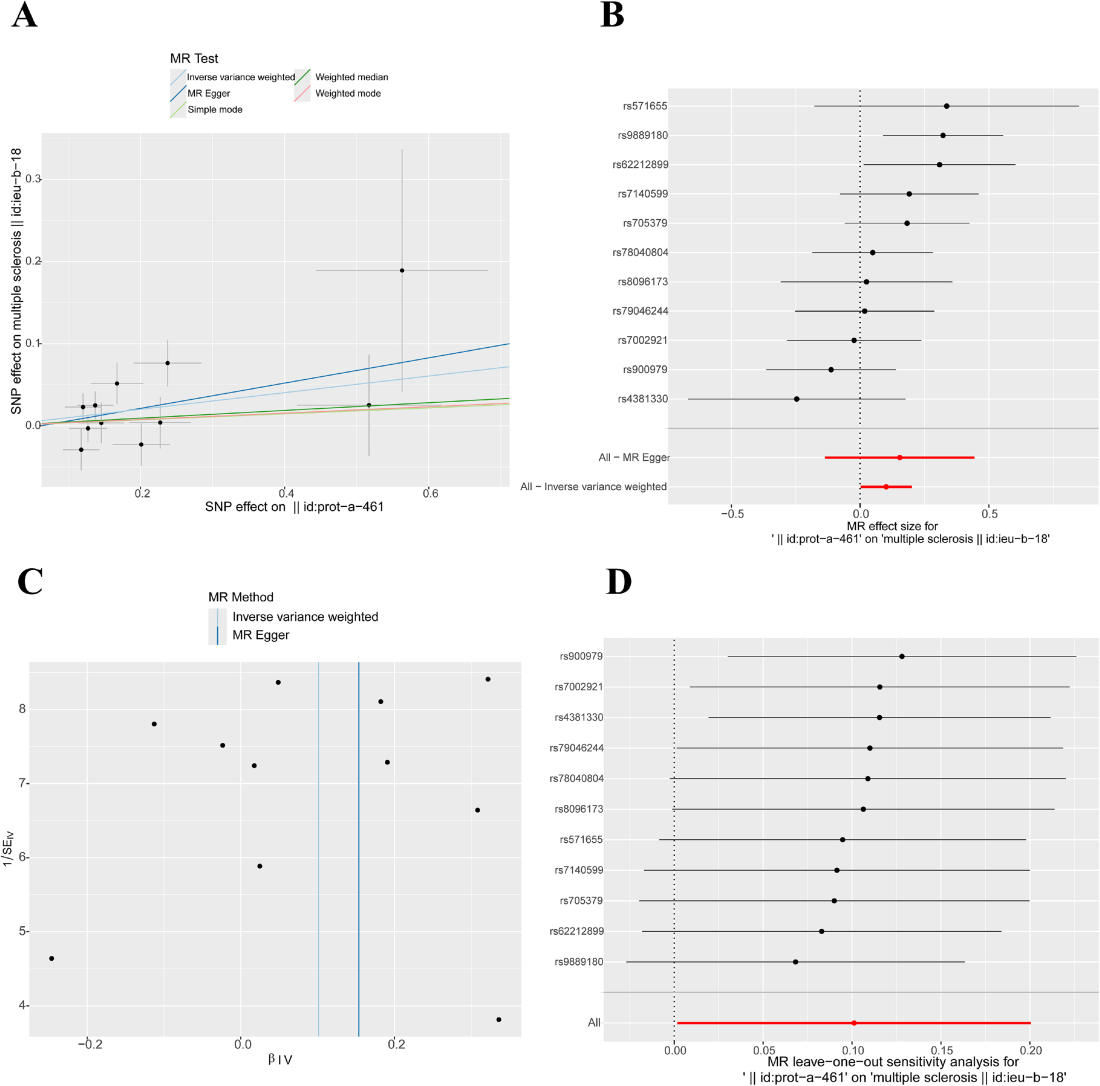
Mendelian randomization study results. **(A)** Scatter plot of MR results for CD79A and MS: A slope > 0 indicates that an increase in CD79A expression is an unfavorable factor for the onset of MS. **(B)** Forest plot illustrating the causal relationship of each SNP with the risk of MS: The bottom red line is on the right side of 0, indicating a positive correlation between CD79A expression and the onset of MS. **(C)** Funnel plot of MR results for CD79A and MS. **(D)** Sensitivity analysis results: The bottom red line is on the right side of 0, indicating that the result is robust.

We used the IVW method to evaluate the causal relationship between 5 hub genes and MS. The research results showed that only CD79A (a total of 11 IVs that met the MR assumptions were screened) had a causal relationship with MS (OR = 1.106, 95% CI 1.002–1.222, *p* = 0.046). The MR-Egger test showed that there was no horizontal pleiotropy in the analysis data of CD79A and MS (*p* > 0.05), as shown in Supplementary Tables S4, S5 and Figures 5A,B. The funnel plot showed that all the included SNPs were basically symmetrical (Figure 5C). The sensitivity analysis results indicated that after removing each SNP, the overall error bars did not change significantly, that is, all the error bars were on the right side of 0, indicating that the results were reliable (Figure 5D). After IVW analysis of the IVs that met the MR assumptions screened for the remaining 4 hub genes, the results were not significant, as shown in Supplementary Table S6.

## 4 Discussion

The MS is an autoimmune disease with a highly complex pathogenesis. To date, the major identified risk alleles, as well as the majority of risk alleles, are associated with immune responses (20–22). The rapid advancement of bioinformatics has provided new insights into the study of various clinical diseases. In this study, we employed bioinformatics approaches to identify hub immune-related genes in MS, aiming to further explore the pathogenesis of the disease and to provide new directions for the early diagnosis and treatment of MS.

In this study, we analyzed gene expression data from MS gene chips in conjunction with immune-related genes, identifying 28 IM-DEGs with strong associations. Enrichment analysis revealed that these genes primarily cluster in pathways related to antigen receptor-mediated signaling, B cell differentiation, B cell proliferation, and B cell receptor signaling. While MS is traditionally regarded as T cell-dominated, an increasing body of research has shown that B cells also play a significant role in MS (23–25). B cells can internalize and process natural antigens, subsequently presenting degraded peptide fragments in association with major histocompatibility complex (MHC) class II molecules to antigen-specific CD4^+^ T cells, thereby functioning as antigen-presenting cells and promoting the pathogenesis of MS. Additionally, B cells can secrete autoantibodies that assist other antigen-presenting cells in presenting antigens related to the central nervous system (26–28). Furthermore, an expanding number of studies have demonstrated that B cell depletion therapy can effectively treat MS and other autoimmune diseases (29). This underscores the importance of these biological functions and signaling pathways in the context of MS.

Through the analysis of the PPI network, we identified several hub genes potentially involved in the pathogenesis of MS, including PTPRC, CD19, CXCL8, CD79A, IL7, CR2, CD22, BLNK, LCN2, and LTF. CD19, CD79A, and CD22 are commonly used pan-B cell markers, with CD19 being the most representative. CD19 is a CD molecule expressed on B cells, and it forms a signaling complex with other B cell surface molecules, such as CR2, CD81, and CD225, to regulate B cell development, proliferation, and differentiation. CD19 is an important therapeutic target in the treatment of diseases such as B cell lymphoma and acute lymphoblastic leukemia (30–32). The ongoing development and refinement of CAR-T therapy have opened new avenues for targeted treatment of MS by specifically targeting CD19-expressing B cells. One study has demonstrated the therapeutic potential of CAR-T cell therapy in late-stage MS patients who are unresponsive to conventional antibody-mediated B cell depletion. Targeting CD19 with CAR-T cells not only suppresses inflammatory relapses but also eliminates residual B cells in the CNS that may contribute to disease progression (33). In addition, during the pathological process of MS, various inflammatory mediators, such as cytokines and chemokines, are activated, which further promote inflammation and demyelination. IL-7, a cytokine belonging to the chemokine family, plays a crucial role in the differentiation, development, and maturation of T and B cells, and is essential for adaptive immunity and the maintenance of immune homeostasis. IL-7 is thought to support the aberrant immune activity associated with MS, although its specific mechanisms remain unclear (34). CXCL8, also known as interleukin-8, can chemotactically recruit and activate neutrophils, mediating the inflammatory response. In mouse models of MS, the expression level of CXCL8 is elevated, facilitating the migration of neutrophils into the CNS and contributing to the progression of the disease (35). Results from nomogram models and ROC curves indicate that PTPRC, CD19, CXCL8, CD79A, and IL-7 exhibit good diagnostic efficacy. The experimental autoimmune encephalomyelitis model is a classic animal model for multiple sclerosis. Therefore, we conducted experiments to validate whether hub genes are differentially expressed between the control group and EAE mice model. Based on the qPCR results, we observed significant differences in the expression of these five hub genes between the control and EAE groups. In summary, the identification of these hub genes provides potential therapeutic targets for MS.

Genetic variations associated with hub genes in MR studies can serve as instrumental variables to infer causal relationships with diseases. Therefore, we conducted an in-depth exploration of the causal relationships between PTPRC, CD19, CXCL8, CD79A, and IL7 with MS through MR analysis. The results indicated that the expression level of CD79A was positively correlated with the incidence of MS. In contrast, the IVW results for the other four genes were not significant, suggesting that their causal relationships are unreliable. CD79A (also known as Igα) serves as a component of the preB cell receptor (preBCR) signaling, forming the BCR complex by non-covalently binding with membrane immunoglobulin (mIg), thereby mediating signal transduction and providing the initial signal required for B cell activation (36). Studies indicate an association between CD79A and central nervous system infiltration, as well as relapse in B cell precursor (BCP) acute lymphoblastic leukemia (ALL) patients (37). In addition, CD79A represents an effective inhibitor target for human B cell activation, rendering it a promising therapeutic option for autoimmune diseases such as systemic lupus erythematosus and collagen-induced arthritis (38, 39). Currently, B cell depletion therapies are a prominent research direction in the field of MS, including CAR-T cell therapy and monoclonal antibody treatments. Among these, B cell-related monoclonal antibodies, regarded as a relatively “mature” approach, have demonstrated favorable efficacy and safety in the treatment of MS. For instance, ocrelizumab, a monoclonal antibody targeting CD20, has been widely used in the treatment of MS and has shown good safety profiles (40, 41). CD79A is another commonly used B cell marker aside from CD20; however, there have been no reported studies on targeting CD79A for the treatment of MS. It is worth noting that in the PCR validation experiment, we observed a significant upregulation of CD79A expression in the EAE group compared to the control group, and this expression trend is consistent with the findings from the MR study. Therefore, it may be worthwhile to explore the potential of CD79A as a therapeutic target for MS, drawing inspiration from the concept of B cell depletion therapies.

This study has certain limitations. First, due to the limited availability of peripheral blood mononuclear cell gene expression data for MS in publicly available datasets, we utilized only one dataset for analysis, which may have led to suboptimal results. Second, the databases used for screening immune gene sets may not be comprehensive, potentially resulting in the omission of some genes. Lastly, this study only performed a preliminary validation of hub gene mRNA levels using qPCR, and further *in vivo* and *in vitro* experiments are needed to confirm their roles and potential mechanisms in MS. In future studies, we will consider verifying the clinical potential of CD79A as a biomarker and a therapeutic target through relevant experiments. For example, cell line experiments on B cells can be conducted to study the role of CD79A in activation, proliferation, and differentiation, as well as to detect the binding affinity of potential drugs. In *in vivo* experiments, the EAE model can be used to evaluate the effect of modulating CD79A through genetic manipulation and pharmacokinetic/pharmacodynamic studies. Additionally, CD79A in peripheral blood mononuclear cells from patients and control groups, as well as tissue samples from affected brain regions, can be analyzed to understand its association with the disease.

## Conclusion

We explored hub immune genes associated with MS using bioinformatics approaches and MR studies. This may provide new insights for the diagnosis and treatment of MS.

## Data Availability

The original contributions presented in the study are included in the article/Supplementary material, further inquiries can be directed to the corresponding author.

## Footnotes

^1^https://www.ncbi.nlm.nih.gov/geo/

^2^https://www.immport.org

^3^http://string-db.org
