# Supplementary material for: Combining gene expression microarrays and Mendelian randomization: exploring key immune-related genes in multiple sclerosis
Source: Front Neurol. 2024 Nov 27;15:1437778. doi: 10.3389/fneur.2024.1437778 (PMC11631747; doi:10.3389/fneur.2024.1437778)
Supplement: Supplementary file 1 [file Table_1.DOCX]

Supplementary Material

**Supplementary Table S1.** Primers of genes.

| **Gene** | **Sequence** |
| --- | --- |
| PTPRC | F : ATGGTCCTCTGAATAAAGCCCA |
|  | R : TCAGCACTATTGGTAGGCTCC |
| CD19 | F : GGAGGCAATGTTGTGCTGC |
|  | R : ACAATCACTAGCAAGATGCCC |
| IL7 | F : TTCCTCCACTGATCCTTGTTCT |
|  | R : AGCAGCTTCCTTTGTATCATCAC |
| CXCL8 | F : CAAGGCTGGTCCATGCTCC |
|  | R : TGCTATCACTTCCTTTCTGTTGC |
| CD79A | F : TCTTCTTGTCATACGCCTGTTTG |
|  | R : GATGTTAGACTGAAGGCTGAACC |
| beta-actin | F : GGCTGTATTCCCCTCCATCG |
|  | R : CCAGTTGGTAACAATGCCATGT |
